# A Framework for Determining the Optimal Vibratory Frequency of Graded Gravel Fillers Using Hammering Modal Approach and ANN

**DOI:** 10.3390/s24020689

**Published:** 2024-01-22

**Authors:** Xianpu Xiao, Taifeng Li, Feng Lin, Xinzhi Li, Zherui Hao, Jiashen Li

**Affiliations:** 1 China Academy of Railway Sciences Co., Ltd., Beijing 100081, China; xiao18388517746@163.com (X.X.); linfeng_tieke@163.com (F.L.); 2 Department of Civil Engineering, Shijiazhuang Tiedao University, Shijiazhuang 050043, China; lixinzhistdu@163.com; 3 Department of Civil Engineering, Central South University, Changsha 410075, China; zheruihao@csu.edu.cn (Z.H.); 224812312@csu.edu.cn (J.L.)

**Keywords:** high-speed railway subgrade, vibration compaction, optimal vibration frequency, key features, *ANN*

## Abstract

To address the uncertainty of optimal vibratory frequency *f_ov_* of high-speed railway graded gravel (*HRGG*) and achieve high-precision prediction of the *f_ov_*, the following research was conducted. Firstly, commencing with vibratory compaction experiments and the hammering modal analysis method, the resonance frequency *f*_0_ of *HRGG* fillers, varying in compactness *K*, was initially determined. The correlation between *f*_0_ and *f_ov_* was revealed through vibratory compaction experiments conducted at different vibratory frequencies. This correlation was established based on the compaction physical–mechanical properties of *HRGG* fillers, encompassing maximum dry density *ρd*_max_, stiffness *K_rd_*, and bearing capacity coefficient *K*_20_. Secondly, the gray relational analysis algorithm was used to determine the key feature influencing the *f_ov_* based on the quantified relationship between the filler feature and *f_ov_*. Finally, the key features influencing the *f_ov_* were used as input parameters to establish the artificial neural network prediction model (*ANN-PM*) for *f_ov_*. The predictive performance of *ANN-PM* was evaluated from the ablation study, prediction accuracy, and prediction error. The results showed that the *ρ_d_*_max_, *K_rd_*, and *K*_20_ all obtained optimal states when *f_ov_* was set as *f*_0_ for different gradation *HRGG* fillers. Furthermore, it was found that the key features influencing the *f_ov_* were determined to be the maximum particle diameter *d*_max_, gradation parameters *b* and *m*, flat and elongated particles in coarse aggregate *Q_e_*, and the Los Angeles abrasion of coarse aggregate *LAA*. Among them, the influence of *d*_max_ on the *ANN-PM* predictive performance was the most significant. On the training and testing sets, the goodness-of-fit *R*^2^ of *ANN-PM* all exceeded 0.95, and the prediction errors were small, which indicated that the accuracy of *ANN-PM* predictions was relatively high. In addition, it was clear that the *ANN-PM* exhibited excellent robust performance. The research results provide a novel method for determining the *f_ov_* of subgrade fillers and provide theoretical guidance for the intelligent construction of high-speed railway subgrades.

## 1. Introduction

The compaction quality control of high-speed railway graded gravel (*HRGG*) was a crucial factor influencing the service performance of the subgrade [1,2,3]. Vibratory compaction was the mainstream method in subgrade construction, where the vibration frequency was closely related to compaction quality control. Unreasonable vibration frequencies could lead to poor control of subgrade compaction quality, which caused various types of subgrade diseases, such as uneven settlement [4,5] and permanent deformation [6,7]. Meanwhile, there has been limited research on the intelligent prediction of the optimal vibratory frequency *f_ov_* for vibratory compaction, which has hindered the development of intelligent subgrade construction [8]. Hence, proposing a method for determining the subgrade compaction *f_ov_* and achieving intelligent prediction of the compaction *f_ov_* are of great guiding significance for improving the service performance of the subgrade and developing intelligent construction for high-speed railway subgrades.

Existing research highlights a robust correlation between vibration frequency and the dry density *ρ_d_* of coarse-grained soil fillers. Through plate vibration compaction experiments, Wang et al. [9] and Ji et al. [10] identified an optimal vibration frequency range (25~27 Hz) under varying excitation forces, resulting in the attainment of maximum dry density during the compaction of coarse-grained soil fillers. Moreover, in vibration compaction experiments, Xie et al. [11] observed that employing the optimal frequency reduces *HRGG* filler crushing, contributing to its optimal mechanical performance. Furthermore, based on indoor experiments, Ye et al. [12] found that when the vibration frequency (25~30 Hz) approaches the resonance frequency of the fillers, the structure of coarse-grained soil fillers becomes more compact, resulting in the maximum dry density. It was observed that there existed an optimal vibration frequency *f_ov_* within coarse-grained fillers during vibratory compaction, which resulted in the optimal compaction state of the fillers. Additionally, the study indicates a close relationship between the resonance frequency *f*_0_ of the fillers and the optimal vibration frequency [13,14]. Furthermore, to improve the efficiency of vibratory compaction for coarse-grained fillers, many scholars conducted research on determining the *f_ov_* for coarse-grained fillers. Xie et al. [15] concluded that the *f*_0_ of coarse-grained soil fillers increased with compaction density. Additionally, it was observed that the compaction performance of the fillers was optimal when the vibration frequency matched the *f*_0_. Hence, it was urgent to investigate the relationship between *f*_0_ and compacted *f_ov_*, and propose a new method to determine the *f_ov_*.

With the development of intelligent compaction technology, the high-precision prediction of vibratory compaction parameters became a crucial part of intelligent subgrade construction [16,17]. Recently, many scholars have established the relationship between compaction parameters and filler features using linear regression models [18]. Nevertheless, there was a clear non-linear relationship between *f_ov_* and filler characteristics, and the accuracy of this model was still open to question. In previous studies, machine learning (*ML*), recognized for its non-linear mapping capability, has proven to be an effective approach for predicting parameters in vibratory compaction. For example, Isik [19] applied the artificial neural network (*ANN*) algorithm to forecast compaction parameters in fine-grained soil and substantiated the suitability of the *ANN* algorithm by utilizing accuracy and error metrics such as goodness-of-fit (*R*^2^) and mean square error (*MSE*). Zaman et al. [20] established an *ANN* model to quantify the relationship between the elastic modulus and stress state of roadbed-graded aggregates. Additionally, Xie et al. [21] found that the *ANN* model had a better predictive capability for the optimal moisture content of *HRGG* fillers during vibratory compaction. All the above studies employed the *ANN* model to predict vibratory compaction parameters, and the prediction results were favorable, which indicated that the *ANN* model exhibited a strong predictive capability for vibratory compaction parameters. Additionally, considering the multitude of factors influencing *f_ov_*, taking the key feature influencing the *f_ov_* as input features for the prediction model could reduce the sample space dimension, enhance the predictive performance of the model, and further improve prediction accuracy [22,23]. Nevertheless, there was a lack of systematic characterization of the performance of coarse-grained fillers, especially the relationship between gradation, particle shape, particle crushing, and *f_ov_*. Hence, it was necessary to determine the key feature influencing the *f_ov_*.

In summary, to address the issue of uncertainty in the vibration compaction *f_ov_* of *HRGG* fillers and achieve intelligent prediction of *f_ov_*, this paper conducts the following research. Firstly, based on vibratory compaction experiments and the hammering modal analysis method, the *f*_0_ of different compaction degrees *K* of fillers was determined. Furthermore, the correlation between *f*_0_ and *f_ov_* was revealed based on compaction experiments at different vibratory frequencies and based on the maximum dry density *ρd*_max_, dynamic stiffness *K_rd_*, and bearing capacity coefficient *K*_20_ of the fillers. Secondly, the gray relational analysis algorithm was used to determine the key feature influencing the *f_ov_* based on the quantified relationship between the filler feature and *f_ov_*. Finally, the key features influencing the *f_ov_* were used as input parameters to establish the artificial neural network prediction model (*ANN-PM*) for *f_ov_*, and the predictive performance of *ANN-PM* was evaluated from the prediction accuracy and error. This research not only determined the *f_ov_* of HRGG fillers through the hammering modal analysis method, but also achieved intelligent prediction of *f_ov_* based on the *ANN* model. This provides a theoretical foundation for intelligent construction in high-speed railway subgrades.

## 2. The Method of Determining *f_ov_*

### 2.1. Material

**Figure 1:**
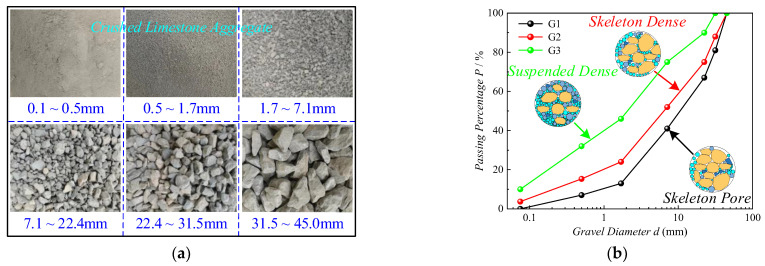
Experimental material: (**a**) crushed limestone aggregate, (**b**) three typical gradation curves.

As shown in Figure 1a, the *HRGG* fillers used in this experiment were surface subgrade fillers of the high-speed railway, which consisted of crushed limestone gravel. Based on the Code for Railway Subgrade Design (TB 10001-2016) [24], the gradation of fillers should meet the following requirements: the maximum particle diameter *d*_max_ ≤ 60 mm, the coefficient of uniformity *C_u_* ≥ 15, and the coefficient of curvature *C_c_* = 1~3. Hence, as shown in Figure 1b, three types of gradation were selected as experimental fillers: skeleton pore gradation G1 (*d*_max_ = 45 mm, *C_u_* = 18.2, *C_c_* = 1.374), skeleton dense gradation G2 (*d*_max_ = 45 mm, *C_u_* = 40.0, *C_c_* = 2.243), and suspended dense gradation G3 (*d*_max_ = 40.5 mm, *C_u_* = 53.333, *C_c_* = 1.2).

### 2.2. Experimental Design

**Figure 2:**
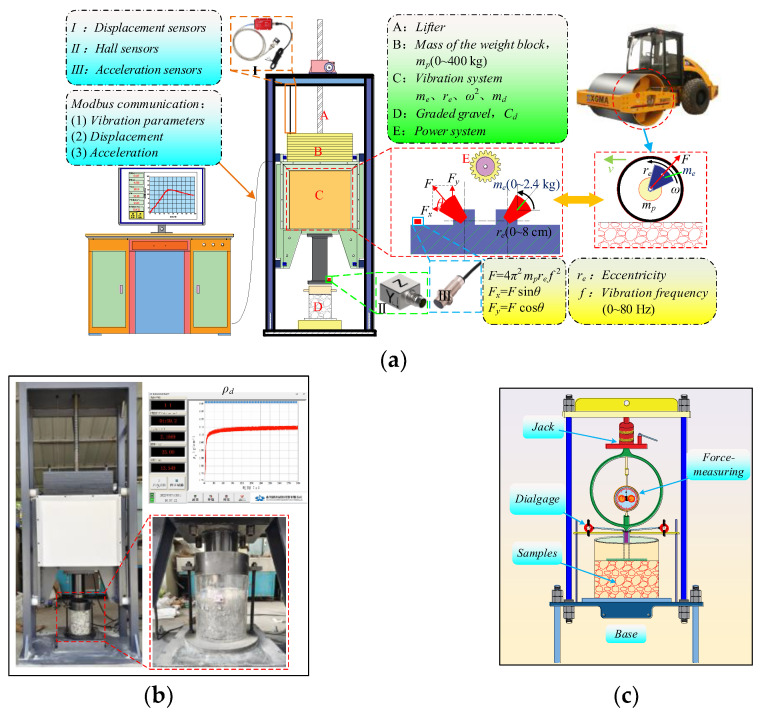
Experimental equipment: (**a**) intelligent compaction equipment, (**b**) experimental data collection, and (**c**) indoor flatbed loading equipment.

Vibratory compaction experiments were conducted using an improved large-scale intelligent vibratory compactor [15]. The equipment was not only equipped with four adjustable parameters, containing the vibratory frequency *f* (0~80 Hz), static load *m_p_* (0~400 kg), eccentricity distance *r_e_* (0~8 cm), and eccentric mass *m_e_* (0~2.4 kg), but also its compaction mechanism was similar to the on-site roller compaction. As shown in Figure 2a, it was important to note that compared with the conventional vibratory compactor, this equipment embedded displacement sensors, hall sensors, and vibratory acceleration sensors. Furthermore, based on Equations (1) and (2), the real-time output of the dry density *ρ_d_* curve and dynamic stiffness *K_rd_* curve can be obtained.

(1) $$\rho_{d}=\frac{mg}{\pi D_{c}^{2}\left(h_{0}-S_{n}\right)}$$

(2) $$K_{rd}=\frac{m_{e}r_{e}\omega^{2}\sin\left(\Delta \varphi \right)+m_{p}g-{m_{d}\ddot{x}|}_{\dot{x}=0}}{{x|}_{\dot{x}=0}}$$

where *m* is the mass of fillers; *D_c_* is the internal diameter of the compaction cylinder; *h*_0_ is the pavement thickness; *S_n_* is the displacement rate of fillers; *m_e_* is the mass of the eccentric block; *r_e_* is the eccentricity; *ω* is the rotation speed of the eccentric block; ∆*φ* is the lag phase angle, which is obtained from the hall sensor; *m_p_* is the mass of the weight block; *m_d_* is the mass of the vibratory system; *x* is the displacement of the vibratory system, which is obtained from the displacement sensors; *ẍ* is the acceleration of the vibratory system, which is obtained from the acceleration sensors.

Applying the method proposed by Xie et al. [25] to determine the maximum dry density *ρd*_max_, the compaction degree *K* of *HRGG* fillers can be calculated in real time by Equation (3). Hence, as shown in Figure 2b, the fillers with different *K* can be accurately obtained by controlling the vibratory compaction time. Additionally, as shown in Figure 2c, to validate the efficacy of the *K_rd_* obtained from the intelligent vibratory compactor, the bearing capacity coefficient *K*_30_ was used to evaluate the mechanical properties of the fillers. To ensure consistency between the indoor *K*_30_ and field experiments, it is necessary to scale down the *K*_30_ of the field using the theory of similarity. The *K*_30_ in this experiment was computed by Equation (4), which was derived based on the similarity coefficient for *K*_30_ proposed by Xie et al. [11].

(3) $$K=\frac{\rho_{d}}{\rho_{d\max}}$$

(4) $$K_{30}=K_{20}=\frac{\sigma_{0.84}}{S_{0.84}}$$

where *S*_0_._84_ is a sinkage of 0.84 mm and *σ*_0_._84_ is the load strength corresponding to a sinkage of 0.84 mm.

To improve the compaction quality and control the particle crushing, the parameters for the vibratory compaction experiments were selected using the optimal parameter determination method based on the resonance frequency *f*_0_ proposed by Xie et al. [25]. The vibratory frequency *f* was set to the *f*_0_ of the fillers, the excitation force *F*_0_/*m_p_* < 1.9, the moisture content *ω* was set to the critical moisture content of the fillers, the diameter-to-diameter ratio (*D_c_*/*d*_max_) was set to 3.9, and the thickness-to-diameter ratio (*h*_0_/*d*_max_) was set to 3.5. Hence, in this paper, the parameters for the vibratory compaction experiments were shown in Table 1.

**Figure 3:**
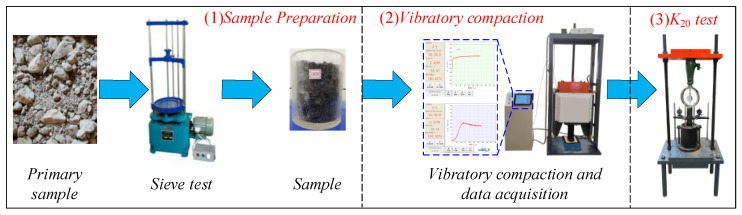
The diagram of vibratory compaction experiments.

As shown in Figure 3, the vibratory compaction experiments were primarily divided into three steps. (1) Sample preparation: the initial fillers were classified based on particle size through sieving tests, and the samples were prepared according to the experimental gradation. (2) Vibratory compaction: experiments were conducted using the intelligent vibratory compactor, and the ρd and *K_rd_* of the *HRGG* fillers were collected in real time. (3) *K*_20_ testing: the *K*_20_ of the *HRGG* fillers after vibratory compaction was tested based on a plate load test.

### 2.3. The Tests of Determining f_0_

**Figure 4:**
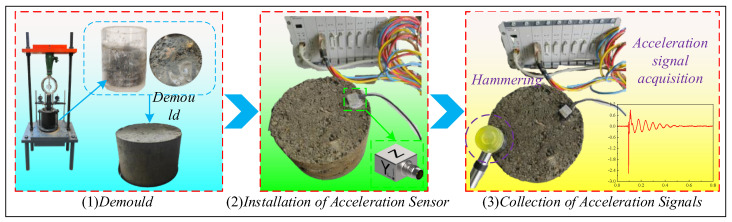
The diagram of hammer impact experiments.

The post-compaction *f*_0_ of the *HRGG* fillers was obtained from the hammering modal analysis method [26]. As shown in Figure 4, the hammering modal experiments were primarily divided into three steps. (1) Demold: the complete compacted HRGG fillers were obtained using demolding equipment after the plate load test. (2) Installation of acceleration sensors: a triaxial accelerometer was installed at the top of the fillers and connected to the DH5922D dynamic signal acquisition equipment. (3) Collection of hammer impact acceleration signals: a rubber hammer was used to strike the top of the fillers, and the acceleration signals of the fillers during the strike were recorded in real time. To ensure the reliability of the signals, the hammering modal experiments for fillers with different *K* were repeated three times. Based on the acceleration signals of the fillers, the f_0_ with different *K* of fillers was determined by the hammering modal analysis method.

**Figure 5:**
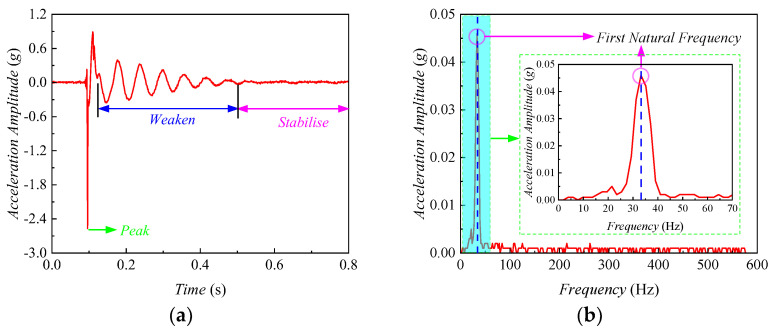
Hammer impact modal analysis: (**a**) acceleration time-domain amplitude, (**b**) acceleration amplitude-frequency spectrum.

Figure 5 shows the hammering modal method analysis process based on G2 type *HRGG* fillers. Figure 5a shows the time-domain amplitude of the acceleration signal. The acceleration amplitude gradually weakened after reaching the peak until stabilized, indicating the fillers’ vibratory feature during the strike. The initial peak indicated a rapid response of the fillers to the hammering, while the weakening process indicated that the hammering energy gradually dissipated within the fillers until stabilized. As shown in Figure 5b, the time-domain acceleration signal was subjected to Fourier transformation to obtain the acceleration signal spectrum. It was observed that the acceleration reached the peak at a frequency of 33 Hz, which indicated a significant vibratory response of the fillers at this frequency. Hence, 33 Hz was determined as the first *f*_0_ of the G2 type *HRGG* fillers.

**Figure 6:**
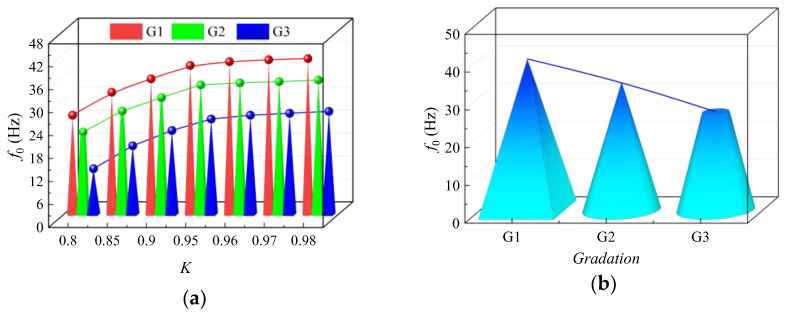
Relationship between *K*, gradation, and *f*_0_: (**a**) relationship between *K* and *f*_0_, (**b**) relationship between gradation and *f*_0_ when *K* = 0.96.

As shown in Figure 6, hammering modal experiments were conducted on *HRGG* fillers with different *K* and gradation, revealing the relationship between grading, *K*, and *f*_0_. As shown in Figure 6a, with the *K* increasing, the *f*_0_ of all three fillers showed a pattern of “rapid increase—slower increase”, and when *K* > 0.95, *f*_0_ tended to stabilize. As shown in Figure 6b, when *K* = 0.96, the filler gradation transitioned from G1 to G3, and the coarse particle content gradually decreased, which led to a gradual reduction in *f*_0_.

### 2.4. Relationship between f_o_ and f_ov_

In summary, the *f*_0_ of *HRGG* fillers with different *K* had been determined by hammering modal experiments. Vibratory compaction experiments were conducted with vibratory frequencies set at 20, 25, 30, 35, 40, and 45 Hz. Furthermore, the relationship between *f*_0_ and *f_ov_* was explored by the parameters *K_rd_*, *K*_20_, and *ρ_d_* of the fillers.

**Figure 7:**
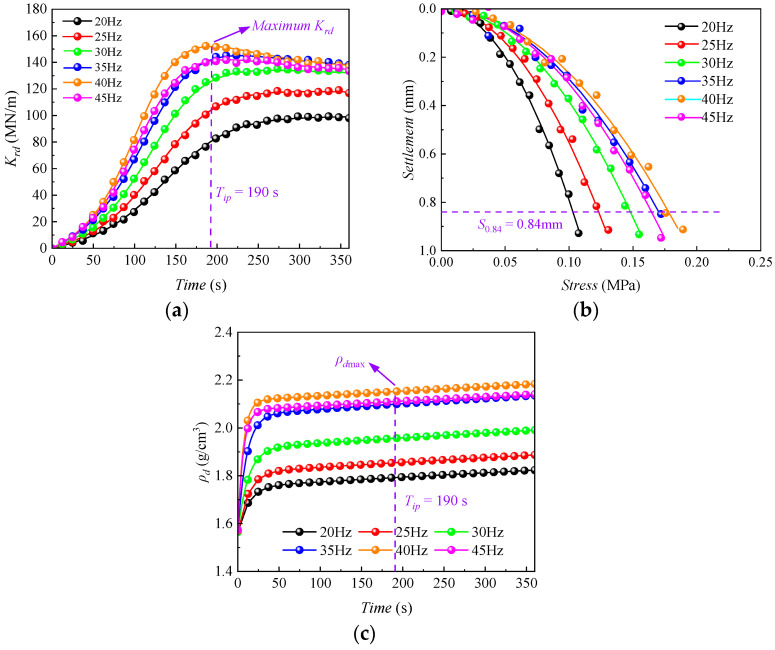
Evolution of *K_rd_*, *K*_20_, and *ρ_d_* of graded gravel (G1) under different vibratory frequencies: (**a**) *K_rd_* time history curve, (**b**) *K*_20_ time history curve, (**c**) *ρ_d_* time history curve.

As shown in Figure 7, the evolution patterns of *K_rd_*, *K*_20_, and *ρ_d_* for the *HRGG* fillers under different vibratory frequencies during the vibratory compaction were obtained. As shown in Figure 7a, the *K_rd_* of fillers at different vibratory frequencies all exhibited a pattern of “rapid increase—slower decrease”, indicating the presence of an “inflection point” in the *K_rd_* curve. As shown in Figure 7b, when the vibratory frequency was set as *f*_0_, the *K*_20_ also reached the maximum value at the “inflection point” of *K_rd_*. As shown in Figure 7c, the *ρ_d_* of fillers at different vibratory frequencies all showed a pattern of “rapid increase—slower increase”. Nevertheless, it was difficult to determine the maximum dry density *ρ_d_*_max_ based on the evolution pattern of *ρ_d_*. Hence, *ρ_d_*_max_ could be determined on the *ρ_d_* curve by the vibratory time *T_ip_* corresponding to the “inflection point” on the *K_rd_* curve [25].

**Figure 8:**
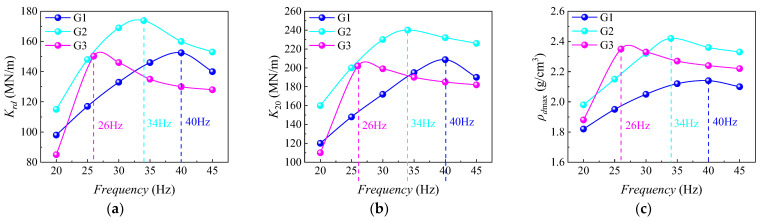
Relationship between vibratory frequency and maximum *K_rd_*, *K*_20_, and *ρ_d_*_max_: (**a**) relationship between vibratory frequency and maximum *K_rd_*, (**b**) relationship between vibratory frequency and *K*_20_, and (**c**) relationship between vibratory frequency and *ρ_d_*_max_.

As shown in Figure 8, the relationships between *K_rd_*, *K*_20_, and *ρ_d_*_max_ of *HRGG* fillers, in relation to vibratory frequency and gradation, were obtained from vibratory compaction experiments. As shown in Figure 8a, when the vibratory frequency was *f*_0_, fillers with different gradations all exhibited the maximum *K_rd_*. Similarly, as shown in Figure 8b,c, when the vibratory frequency was *f*_0_, the *K*_20_ and *ρ_d_*_max_ all reached maximum values, which was consistent with the evolution patterns of *K_rd_*. The above experimental results indicated that the mechanical and physical properties of the compacted fillers were optimal when the vibratory frequency was *f*_0_, further indicating that *f*_0_ was the optimal compaction frequency.

## 3. ANN-Based Predictive Model for *f_ov_*

### 3.1. Key Feature of f_ov_

**Figure 9:**
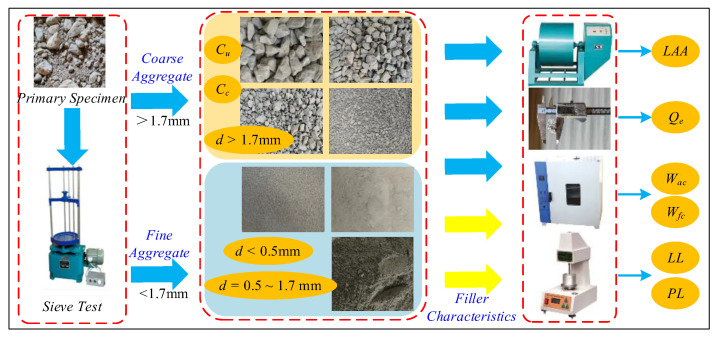
Performance feature experiments of fillers.

According to the Chinese Code for Design of Railway Earth Structure (TB 10001–2016) [1], the performance feature of the fillers include gradation, particle shape, and particle crushing, such as *C_u_*, *C_c_*, *d*_max_, three typical particle diameters (*d* ≤ 0.5 mm, *d* = 0.5~1.7 mm, *d* ≥ 1.7 mm), the Los Angeles abrasion of coarse aggregate *LAA*, flat and elongated particles in coarse aggregate *Q_e_*, the water absorption of coarse aggregate *W_ac_*, the water absorption of fine aggregate *W_af_*, the liquid limit of fine aggregate *LL,* and the plastic limit of fine aggregate *PL*. As shown in Figure 9, to identify the feature of influencing the *f*_ov_, all features were tested in the vibratory compaction experiments based on the Railway Ballast (TB/T 2140-2008) [27] and Geotechnical Testing Procedures for Railway Engineering (TB 10102-2023) [28].

Recently, the relationship between all features and *f_ov_* was still unclear. If all features influencing *f_ov_* were inputted into the *ML* prediction model, this might have overshadowed the role of key features and increased the difficulty of model training. Hence, it was necessary to identify the key features influencing *f_ov_*, and then input them into the *ML* prediction model to reduce the spatial dimension of the samples and enhance the efficiency of model training.

**Figure 10:**
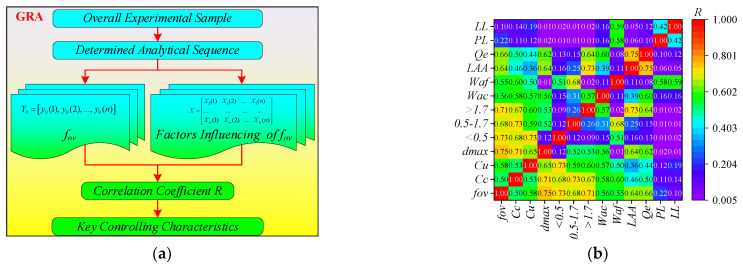
Based on the GRA algorithm analysis of *f_ov_* key characteristics: (**a**) flowchart of GRA algorithm, (**b**) characterization analysis results.

As shown in Figure 10a, Grey Relational Analysis (GRA) was a statistical method for analyzing multiple factors, which assessed the correlation of sequences based on the similarity of their curve shapes. The similarity in sequence curve shapes was positively correlated with sequence correlation [29]. Hence, the key feature of influencing the *f_ov_* could be determined based on *GRA*. As shown in Figure 10b and Table 2, the correlation coefficient *R* between each characteristic and *f_ov_* was calculated. Generally, the feature could be considered as strongly correlated when *R* > 0.6. Thus, the feature strongly correlated with *f_ov_* was as follows: *d*_max_ (0.75), *d* < 0.5 mm (0.73), *d* ≥ 1.7 mm (0.71), *d* = 0.5 mm~1.7 mm (0.68), *Q_e_* (0.66), *LAA* (0.64).

### 3.2. Dataset of ANN Model

The *GRA* algorithm explicitly determined the key feature influencing the *f_ov_*. Nevertheless, it was difficult to provide a detailed description of the gradation feature for *d* ≥ 1.7 mm, *d* = 0.5 mm~1.7 mm, and *d* < 0.5 mm, making it impractical for direct application in the *ML* prediction model. Hence, it was crucial to accurately quantify the gradation feature of the fillers. As shown in Equation (5), Wu et al. [30] proposed an equation that could describe the continuous gradation of coarse-grained soil. Thus, the three indicators of the gradation feature could be described by the two gradation characteristic parameters: b and m. Eventually, *d*_max_, *b*, *m*, *Q_e_*, and *LAA* were considered as the key features of influencing the *f_ov_* and were used as input features for the *ML* prediction model.

(5) $$P=\frac{1}{\left(1-b\right){\left(\frac{d_{\max}}{d}\right)}^{m}+b}\times 100\%$$

where *b* and *m* are the gradation characteristic parameters.

**Figure 11:**
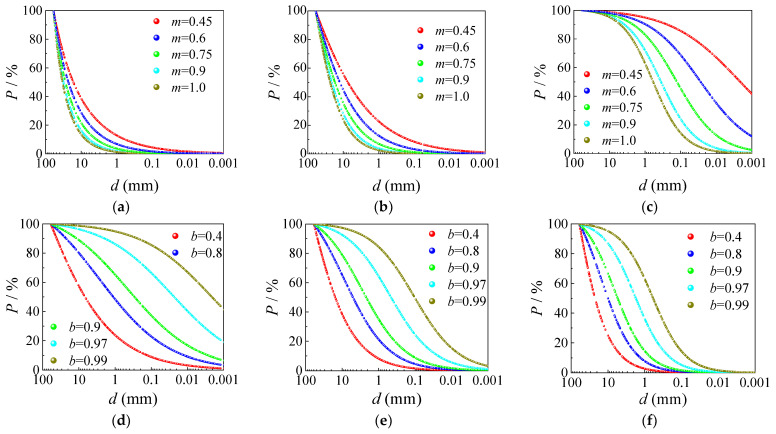
Relationship between gradation characteristic parameters and curve shape: (**a**) *b* = −0.28, (**b**) *b* = 0.36, (**c**) *b* = 1.0, (**d**) *m* = 0.45, (**e**) *m* = 0.725, and (**f**) *m* = 1.0.

To validate the reasonableness of Equation (5) in describing the gradation feature of *HRGG* fillers, as shown in Figure 11, the *d*_max_ was set to 60 mm, and different values were assigned to *b* and *m*, resulting in gradation curves of different forms. The slope of the gradation curve was primarily determined by *m*. When *b* was held constant, *m* was positively correlated with the slope of the gradation curve. Meanwhile, the shape of the gradation curve was mainly determined by *b*. When *m* was held constant, with the *b* increasing, the gradation curve gradually transitioned from “hyperbolic” to “reverse S-shaped”. In conclusion, the parameters *m* and *b* in the gradation equation, respectively determined the slope and shape of the gradation curve. The wide range of variation in the *b* and *m* allowed the gradation equation to reflect different forms of gradation curves. Hence, it was reasonable to use Equation (5) to describe the gradation feature of *HRGG* fillers.

**Figure 12:**
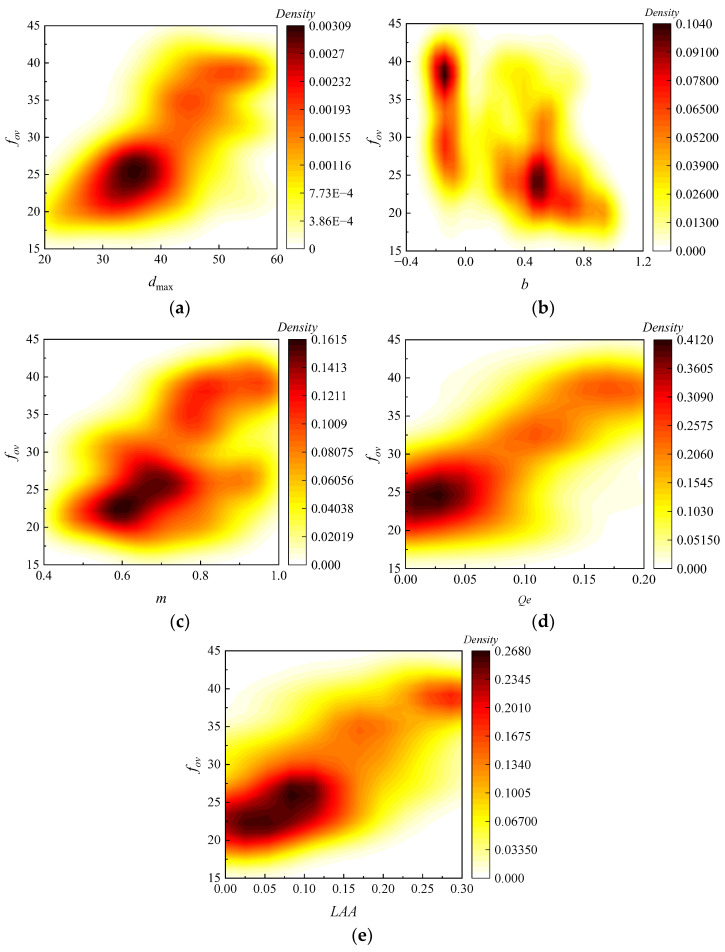
Relationship between the key features and *f_ov_*: (**a**) *d*_max_, (**b**) *b*, (**c**) *m*, (**d**) *Q_e_*, and (**e**) *LAA*.

The *ML* prediction model dataset was further constructed based on the five key features of influencing the *f_ov_*. A dataset *D* = {(*x_ρ_*, *y_ρ_*)}*_N_* _*i*=1_ was established by analyzing the relationship between *f_ov_* and the five key features for 333 sets of fillers with different gradations, where *x_ρ_* represented the input feature consisting of the five key characteristics, and *y_ρ_* represented the output feature consisting of *f_ov_*. As shown in Figure 12, the relationships between the five key features and *f_ov_* in the *ML* prediction model dataset were obtained by analysis.

### 3.3. Establishment and Evaluation of ANN-PM

**Figure 13:**
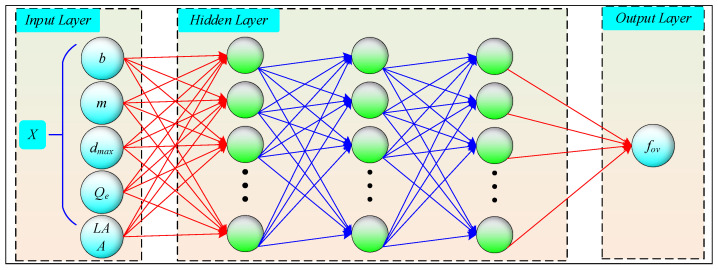
Architecture of artificial neural network.

**Figure 14:**
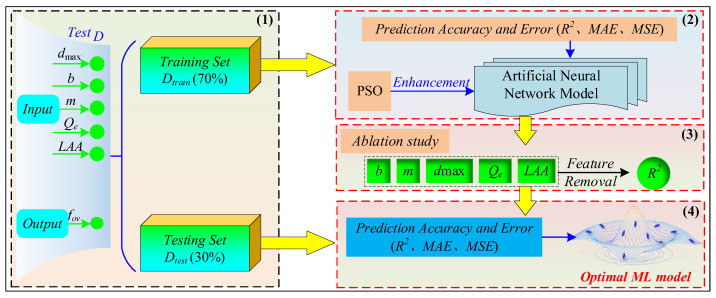
Schematic of *ANN*-based *f_ov_* prediction model.

As shown in Figure 13, Artificial Neural Network (ANN) was a typical *ML* algorithm [31,32,33,34,35,36] used for predictive analysis of the *f_ov_*. Additionally, the *ANN* model was trained using the Adam optimizer algorithm [37,38]. During the training, the mean absolute error (*MAE*) was used as the objective function to calculate particle fitness, which could be calculated by Equation (6). As shown in Figure 14, the establishment of the *ANN* prediction model (*ANN-PM*) based on *f_ov_* primarily involved three steps: (1) partitioning the dataset, (2) developing the *ANN-PM* based on the training set, (3) and evaluating the performance of the *ANN-PM* based on the testing set.

(6) $$fitness=MAE=\frac{1}{n}\sum_{i=1}^{1}\left|y_{i}-{\hat{y}}_{i}\right|$$

where *n* is the sample size, *y_i_* is the true value, and *ŷ_i_* is the predicted value.

**Step 1: Partitioning the dataset.**

The dataset *D* was divided into training and testing sets in a 7:3 ratio, where the training set was used to develop the *ML* prediction model, and the testing set was used to evaluate the performance of the *ANN-PM*.

**Step 2: Developing the *ANN-PM* based on the training set.**

The *ANN* model was employed to predict *f_ov_*, while the *particle swarm optimization* (*PSO*) algorithm [39,40] was introduced to optimize the hyperparameters of the *ANN-PM*. This method has been confirmed as an effective approach for hyperparameter optimization [41]. After that, the training set was inputted into the *ANN-PM*, and it was trained based on the optimal hyperparameters. The goodness-of-fit *R*^2^ [42], mean square error (*MSE*) [43], and mean absolute error (*MAE*) [44] were used to evaluate the generalization ability of the *ANN-PM*.

**Step 3: Evaluating the impact of key features on the *ANN-PM* performance based on the ablation study.**

A series of prediction experiments was designed by removing key features to explore their impact on the *ANN-PM* predictive performance [45]. Similarly, *R*^2^ was employed to evaluate the predictive performance of *ANN-PM* under different experimental conditions.

**Step 4: Evaluating the performance of the *ANN-PM* based on the testing set.**

After the *ANN-PM* was developed, the testing set was used for *f_ov_* prediction. To quantify the generalization ability of the *ANN-PM*, the same evaluation criteria (*R*^2^, *MAE*, and *MSE*) for prediction accuracy and error as in step 2 were applied to evaluate the predictive performance of the *ANN-PM*.

### 3.4. Sensitivity Analysis of ANN-PM

**Figure 15:**
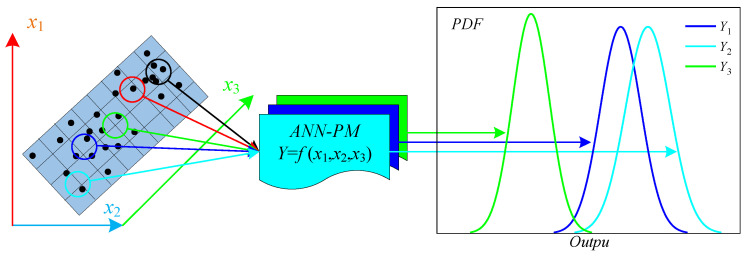
Schematic of the Monte Carlo method.

As shown in Figure 15, based on the Monte Carlo method, the random fluctuations in *ANN-PM* input data could propagate through the output solution [46,47]. Then, the quantitative analysis of the probability distribution of the output solution could characterize the robustness of the *ANN-PM*.

To comprehensively evaluate the *ANN-PM* performance, the Monte Carlo method was employed to simulate the probability distributions of *R*^2^ and *MSE*. This analysis aimed to evaluate the robustness of the predictive models. The specific steps were outlined as follows:

**Step 1: Randomization of data and result computation.**

Randomizing the training set involves randomly combining 70% of the data for training and making predictions on the testing set. A set of *M*−*R*^2^ and *M*−*MSE* was calculated based on the predicted results, as shown in Equations (7) and (8).

(7) $$M-R^{2}=f_{R^{2}}(x_{1},x_{2},\ldots x_{n})$$

(8) $$M-MSE=f_{MSE}(x_{1},x_{2},\ldots x_{n})$$

where *M*−*R*^2^ and *M*−*MSE* are the *R*^2^ and *MSE* obtained through the Monte Carlo method. *f_R_*_2_() and *f_MSE_*() denote the non-linear functions between the random input training set, the *R*^2^, and *MSE*.

**Step 2: Repetitive simulation.**

The number of Monte Carlo simulations was set to 300. Next, the computational process outlined in step 1 was repeated, resulting in *N* sets of *M*−*R*^2^ and *M*−*MSE*, which were used to create probability distribution plots for structural analysis.

## 4. Results and Analysis

### 4.1. Establishment of ANN-PM Based on the Training Set

**Figure 16:**
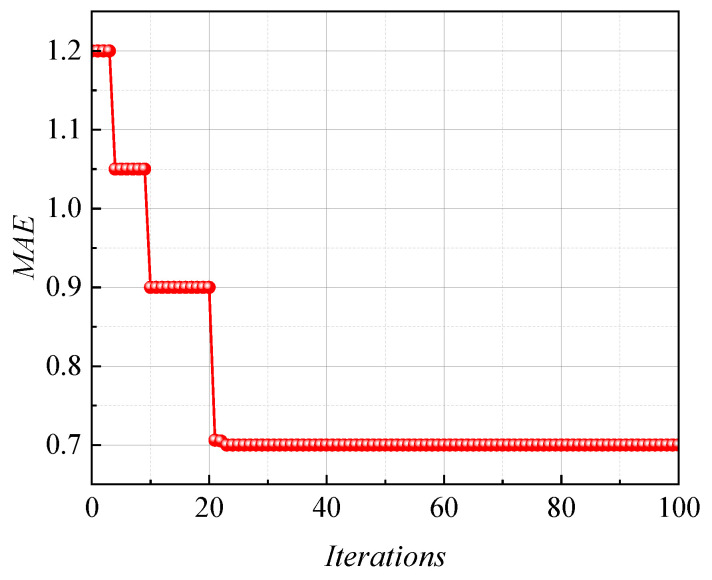
*MAE* values versus some iterations using hybrid models.

Figure 16 shows the variation of fitness for the *ANN-PM* during the iterative. It was clear that, with the iterative optimization of the *PSO* algorithm, the fitness of the *ANN-PM* significantly decreases and tends to stabilize after fewer iterations. This indicated that the *PSO* algorithm has a significant advantage in improving the prediction accuracy of *ANN-PM*. The optimum hyperparameters of the used *ANN*-*PM* in this paper were shown in Table 3. Furthermore, the obtained optimal hyperparameters were inputted into the *ANN-PM* for the prediction of *f_ov_*.

**Figure 17:**
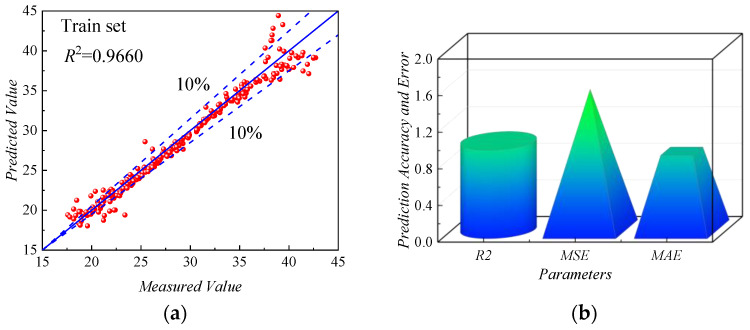
Predictive performance of *ANN-PM* in the training dataset: (**a**) *R*^2^, (**b**) *R^2^*, *MSE*, and *MAE*.

As shown in Figure 17a, the scatter plot showed the fitting results of the *ANN-PM* on the training set, indicating the relationship between the predicted and actual values of *f_ov_*. The horizontal axis represented the actual values of *f_ov_*, while the vertical axis represented the predicted values. The more concentrated the data points were on the 45° median axis, the better fitting capability of the *ANN-MLPM*. The data points of the *ANN-PM* generally fluctuated around the 45° median axis, with the majority of points concentrated within the 10% error range, and only a small number of points fell outside this range, indicating that the *ANN-PM* demonstrated good fitting capability.

As shown in Figure 17b, the predictive performance of the *ANN-PM* on the training set was evaluated from the perspective of prediction accuracy and error. On the training set, the error indicators *MAE* (0.85391 Hz) and *MSE* (1.53176 Hz) of the *ANN-PM* were both small. Additionally, the *R*^2^ of the *ANN-PM* was higher than 0.96, indicating a high level of fitting accuracy. However, the predictive results on the training set only indicated the predictive ability of the *ANN-PM* during the development of the model. Hence, the predictive performance of the *ANN-PM* should have been evaluated using the testing set.

### 4.2. Evaluating the Impact of Key Features on the ANN-PM Performance

**Figure 18:**
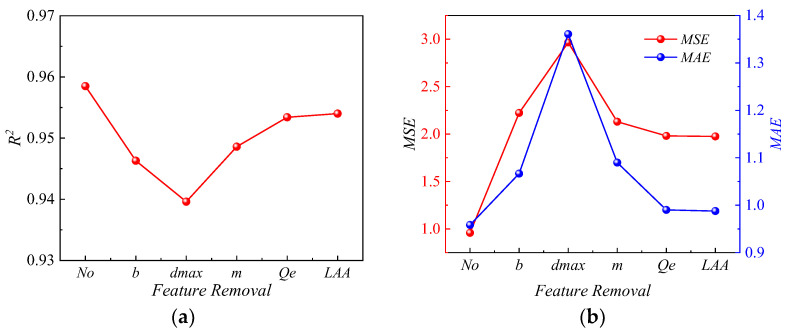
The results of the ablation study: (**a**) *R*^2^, (**b**) *MSE* and *MAE*.

As shown in Figure 18, when five features were used as input features, the *ANN-PM* achieved the highest *R*^2^, indicating the highest prediction accuracy. Subsequently, after removing the *LAA*, the *R*^2^ of the *ANN-PM* was 0.9235, which only decreased by approximately 0.035. This indicated that the importance of the *LAA* for prediction results was relatively low. Conversely, when the *d*_max_ was removed, the *R*^2^ of the *ANN-PM* dropped to 0.8956, falling below 0.9. Similarly, when the *d*_max_ was removed, *MAE* and *MSE* all reached their maximum values. This indicated that the *d*_max_ held significant importance for prediction results. A comparative analysis revealed the importance rankings of the individual metrics as follows: *d*_max_ > *b* > *m* > *Q_e_* > *LAA*.

### 4.3. Evaluation of ANN-PM Based on the Testing Set

**Figure 19:**
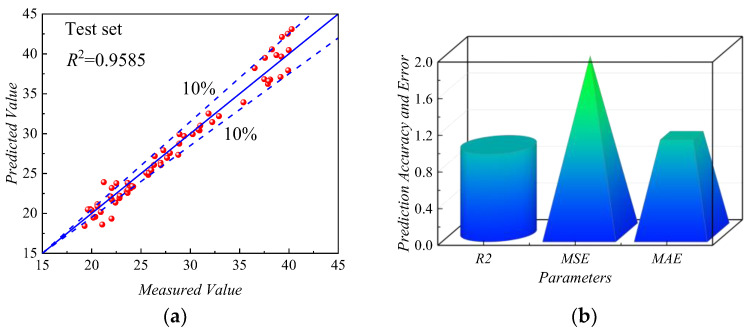
Predictive performance of *ANN-PM* in the test dataset: (**a**) *R*^2^, (**b**) *R^2^*, *MSE*, and *MAE*.

As shown in Figure 19a, the scatter plot showed the fitting results of the *ANN-PM* on the testing set, indicating the relationship between the predicted and actual values of *f_ov_*. The data points of the *ANN-PM* generally fluctuated around the 45° median axis, with the majority of points concentrated within the 10% error range, and only a small number of points fell outside this range, indicating that the *ANN-PM* demonstrated good fitting capability. As shown in Figure 19b, the predictive performance of the *ANN-PM* on the testing set was evaluated from the perspective of prediction accuracy and error. On the testing set, the error indicators *MAE* (1.05942 Hz) and *MSE* (1.93091 Hz) of the *ANN-PM* were both small. Additionally, the *R*^2^ of the *MLPM* was higher than 0.95, indicating a high level of fitting accuracy.

Based on the above, the *ANN-PM* showed good predictive performance in terms of prediction accuracy and error evaluation on both the training and testing sets. Hence, the *ANN-PM* could be employed to predict the *f_ov_* for the vibratory compaction of *HRGG* fillers.

### 4.4. Sensitivity Analysis of ANN-PM

**Figure 20:**
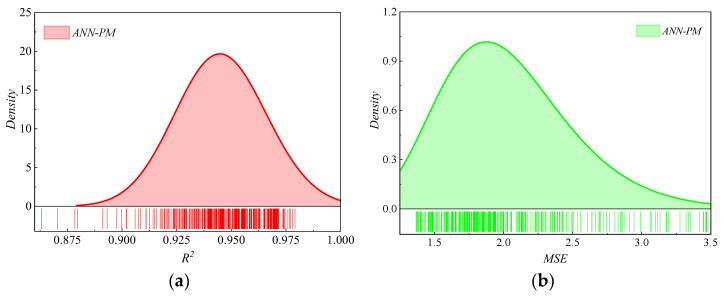
Results of the Monte Carlo method: (**a**) *R*^2^, (**b**) *MSE*.

As shown in Figure 20, the probability distributions of the *R*^2^ and *MSE* were obtained by the Monte Carlo analysis. The distribution of *R*^2^ for the *ANN-PM* closely approached 1, with its mean value exceeding 0.9. This indicated that the *ANN-PM* maintained a high level of predictive accuracy. Simultaneously, the *MSE* distribution for the *ANN-PM* approached zero. Combining the robustness analysis results of *R*^2^ and *MSE*, it was clear that the *ANN-PM* exhibited excellent robust performance.

## 5. Discussion

Based on the results of this paper and the references [11,21,48], it is indicated that the *ANN* model exhibits excellent predictive performance for the vibration compaction parameters (*f_ov_*) of *HRGG* fillers. This highlights the significant advantage of the *ANN* model in *f_ov_* prediction, providing more accurate guidance for practical engineering applications. Hence, in forthcoming *f_ov_* prediction applications, especially in the field of intelligent compaction, the *ANN* model is poised to become a potent tool. As intelligent technologies continue to evolve, this model has the potential to deliver accurate *f_ov_* predictions for practical engineering, further propelling the advancement of intelligent compaction control. Nevertheless, there are still some issues that require further optimization regarding the *ANN* model. Despite demonstrating excellent predictive capabilities, the black-box nature of the *ANN* can hinder its real-world applications due to a lack of transparency in decision making. Hence, an in-depth exploration of data augmentation and model interpretability techniques can enhance the robustness of predictive results. Given these limitations, future research could employ SHapley Additive exPlanations (*SHAP*) analysis to reveal the relative importance of different input features. Moreover, the integration of advanced techniques such as ensemble learning or hybrid models may further improve prediction accuracy and reduce potential uncertainties.

## 6. Conclusions

To address the uncertainty of optimal vibratory frequency *f_ov_* of high-speed railway graded gravel (*HRGG*) and achieve high-precision prediction of the *f_ov_*, the following research was conducted. Firstly, the correlation between the resonance frequency *f*_0_ and *f_ov_* of fillers with different compactness *K* was determined by vibratory compaction experiments and the hammering modal analysis method. Secondly, the relationship between the filler feature and *f_ov_* was established, which revealed the key feature influencing the *f_ov_*. Finally, the artificial neural network prediction model (*ANN-PM*) for predicting *f_ov_* was established based on the key characteristics. The ablation study, prediction errors, and accuracy were used to evaluate the predictive performance of *ANN-PM*. Furthermore, the *ANN-PM* robust performance was evaluated based on the sensitivity analysis. The main conclusions obtained are as follows:

1. In the vibratory compaction experiments, maximum dry density *ρ_d_*_max_, stiffness *K_rd_*, and bearing capacity coefficient *K*_20_ of different gradation *HRGG* fillers all obtained optimal states when the vibratory frequency was set as *f*_0_, which indicated that *f*_0_ was the *f_ov_*.
2. Based on the gray relational analysis algorithm, the key features influencing the *f_ov_* were determined to be the maximum particle diameter *d*_max_, gradation parameters *b* and *m*, flat and elongated particles in coarse aggregate *Q_e_*, and the Los Angeles abrasion of coarse aggregate *LAA*.
3. The key feature influencing the *f_ov_* was used to establish the *ANN-PM*. Then, based on the ablation study, it was indicated that the impact hierarchy of the five key features on the *ANN-PM* predictive performance was *d*_max_ > *b* > *m* > *Q_e_* > *LAA*.
4. On the training and testing sets, the goodness-of-fit *R*^2^ of *ANN-PM* all exceeded 0.95, and the prediction errors were small, which indicated the strong prediction capability of *ANN-PM* for *f_ov_*.
5. Based on the sensitivity analysis, the distribution of *R*^2^ for the *ANN-PM* closely approached 1, with its mean value exceeding 0.9. In addition, the *MSE* distribution for the *ANN-PM* approached zero. It was clear that the *ANN-PM* exhibited excellent robust performance.

## Figures and Tables

**Table 1:** The parameters of vibratory compaction experiments.

Gradation	*f* (Hz)	*ω* (%)	*m_p_* (kg)	*r_e_* (mm)	*m_e_* (kg)	*D_c_* (mm)	*h*_0_ (mm)
G1	40	3.6	600	18.0	4.7	200	155
G2	34	4.0	600	25.1	4.7	200	155
G3	26	5.4	600	44.4	4.7	200	155

**Table 2:** The correlation between different performance features of *HRGG* fillers and *f_ov_*.

Performance feature	*C_u_*	*C_c_*	*d*_max_	*d* ≤ 0.5	*d* = 0.5~1.7	*d* ≥ 1.7	*LAA*	*Q_e_*	*W_ac_*	*W_af_*	*LL*	*PL*
Correlation coefficient *R*	0.58	0.5	0.75	0.73	0.68	0.71	0.64	0.66	0.56	0.55	0.1	0.22

**Table 3:** Optimum hyperparameters of *ANN* models.

Hyperparameters	*α*	Neurons1	Neurons2	Epoch	Batch Size
*ANN*	0.001	100	100	200	16

## Data Availability

Data are contained within the article.
